# Case Report: A 65-year-old man with paraganglioma accompanied by elevated interleukin-6 levels and KIF1B single gene mutation

**DOI:** 10.3389/fendo.2023.1226468

**Published:** 2023-09-14

**Authors:** Chi Wang, Ming Guan, Shuang Zhang, Can Cui

**Affiliations:** Department of Endocrinology and Metabolism, The Second Affiliated Hospital of Harbin Medical University, Harbin, Heilongjiang, China

**Keywords:** paraganglioma, interleukin-6, fever, KIF1B gene, single gene mutation

## Abstract

Paraganglioma is a less prevalent disease, and paraganglioma with only secreting interleukin-6 (IL-6) has not been previously reported. A 64-year-old male patient came to the hospital with the chief complaints of fever and palpitations. The peak body temperature was 38.7°C (101.66°F). Heart rate was 110 bpm, while blood pressure was in the normal range. Antibiotics and antiviral therapies were ineffective. The levels of blood IL-6, C-reactive protein (CRP), alkaline phosphatase (ALP), platelets (PLT), glutamyltransferase (GGT), fibrinogen, and D-dimer were all elevated. Infectious diseases, auto-immune diseases, and hematological malignancy were all excluded. Nearly 10 years ago, a large retroperitoneal mass of the patient was detected by accident. Fortunately, there have been no special symptoms for the past 10 years after regular follow-up. After admission this time, PET-CT was performed. A large confounding density mass at the upper part of the abdominal and retroperitoneal area was seen, and the possibility of paraganglioma was considered. However, biochemical assays for blood and urine catecholamine and their metabolites including adrenaline, norepinephrine, 3-methoxytyramine, methoxyepinephrine, methoxynorepinephrine, and vanillylmandelic acid were all in normal range in spite of mild elevated dopamine with no significance. The whole-exome capture and sequencing of the genomic DNA of the patient showed a heterozygous mutation in the coding site of KIF1B gene (Coding: NM_015047.3:c.4660G>C, Mutation: p.Val1554Leu; chromosomal location was chr1: 10428570). The mutation at this locus of KIF1B has not been reported previously. The patient refused the surgical treatment. Because the mass burdens several important organs including the pancreas, the risk of surgery was high. Doxazosin was then administered to the patient. After taking doxazosin, the symptoms disappeared rapidly. Body temperature returned to normal range in 3 days. Heart rate decreased to approximately 90 bpm. In the following days, the levels of IL-6, CPR, ALP, platelets, GGT fibrinogen, and D-dimer continued to decrease. After 63 days of taking doxazosin, IL-6 level was completely normal. After 190 days of medication, hemoglobin (Hb) and GGT levels also returned to the normal range. After 1 year onset, the patient again underwent a blood test. Almost all blood indexes were in the normal range including IL-6.

## Introduction

Paraganglioma and pheochromocytoma were a family of rare neuroendocrine tumors. They arise from the sympathetic/parasympathetic neural ganglia and chromaffin cells of the adrenal medulla, respectively. The clinical manifestations include hypertension, headache, and palpitations, but owing to different loci, the manifestations are heterogeneous (1–4). Paraganglioma not only diversified its symptoms, but also occurs in various locations. It can appear in the retroperitoneum, duodenum, and even the middle ear (5). In previous case reports, despite the variety of symptoms and different locations of paraganglioma, patients would have a significant increase in catecholamines or their metabolites, along with an elevation of cytokines, including IL-1, IL-6, and TNF-α (6). However, we diagnosed a patient with retroperitoneal paraganglioma with only an increase in serum IL-6 levels without elevated levels of catecholamines and their derivatives. The patient presented only with a monogenic mutation in KIF1B. This patient had only fever and palpitations as the main symptoms, without hypertension and headache. After treatment with doxazosin, the symptoms disappeared and abnormal indicators returned to normal levels in this case report.

## Case presentation

A 65-year-old male patient presented to our hospital due to fever, palpitations, and fatigue in February 2022. The peak body temperature of the patient was 38.7°C. There is no hypertension but tachycardia was present during physical examination at admission time. Blood routine test showed that neutrophilic granulocyte was slightly higher than the normal range. To rule out infectious diseases, bacteria blood cultures, hepatitis, HIV, syphilis, *Brucella*, and COVID-19-related indexes were tested. Chest CT scanning did not give any evidence of respiratory infectious disease. Even high-throughput DNA and RNA sequencing of pathogenic microorganisms was also checked, but no positive results were obtained. Rheumatic and autoimmune diseases were also excluded.

The patient had anemia, and the hemoglobin level was very low. The blood routine showed microcytic hypochromic anemia: 70 g/L↓ (reference range: 110–160 g/L), red blood cell level 2.96 × 10^12^/L↓ (reference range: 2.5–5.5 × 10^12^/L), MCV 79.8 fl↓ (reference range: 80–100 fl), MCH 24.2 pg↓ (reference range: 27–34 pg), and MCHC 304 g/L↓ (reference range: 320–360 g/L). Therefore, after further improving the inspection, it was found that the iron level was 2.5 μmol/L↓ (reference range: 10.6–36.7 μmol/L) and the ferritin level was 472 ng/ml↑ (reference range: 21.81–274.66 ng/ml). Bone marrow image analysis showed proliferative anemia, decreased iron in iron staining, negative bone marrow culture, and no abnormal cells such as plasma cells and hemophagocytic cells, suggesting that the anemia was only caused by chronic wasting disease. Therefore, anemia and fever caused by rare blood diseases such as Castleman disease and HLH were excluded. We also found that the patient’s CRP was significantly elevated: 117 mg/L↑ (reference range: 0–6 mg/L). We tried to give the patient a variety of antibiotics such as cephalosporins, carbapenems, and antiviral drugs, but the patient’s fever did not improve.

The patient’s abdominal ultrasound revealed a large mass in the abdominal cavity. Reviewing the patient’s medical history, because of the routine physical examination, the patient indicated a huge abdominal mass by CT in December 2012, whose size was approximately 105 mm × 68 mm. The patient was hospitalized in the oncology department and interventional department of our hospital in January 2013, and the improved three-dimensional enhanced CT imaging of the abdomen suggested space-occupying lesions in the right abdominal cavity and retroperitoneal area with rich blood supply. The possibility of paraganglioma or vascular lesions was considered. The size was approximately 100 × 140 mm, which seemed to have unclear boundary with the inferior vena cava. The surrounding tissues were compressed and displaced, and there was no indication of lymphadenopathy. At this time, the blood routine, coagulation, and liver function of the patient were normal, and there were no symptoms of fever and palpitation. The patient’s gastroscopy showed that there were nodular bulges in the gastric body, surface erosion, rigidity of the surrounding mucosa, easy bleeding when touching, and narrow gastric cavity in the lesion area. However, pathological examination at the gastric bulge only suggested gastric fundus gland polyps. Because the patient had no obvious discomfort, the paraganglioma volume was very large, multiple organs were compressed, and the operation was high risk, the patient and his family members did not agree to the operation.

In September 2021, the patient was hospitalized in the dermatology department due to a large area of eczema. After treatment with betamethasone injection, he developed systemic edema, dyspnea, and palpitations. Then, the patient was transferred to the cardiology department for diagnosis and treatment. Cardiac color ultrasound suggested that the right chamber of the right atrium was significantly enlarged, with lax tricuspid closure with severe regurgitation. The main pulmonary artery and branches were wider, with pulmonary hypertension (severe). The systolic pressure of pulmonary artery was approximately 96 mmHg, and the right heart function was reduced. The patient’s heart rate was 96 bpm. The blood gas analysis showed a pO_2_ level of 54 mmHg (reference range: 80–100 mmHg) and a pCO_2_ level of 34 mmHg (reference range: 35–45 mmHg), which was type I respiratory failure. Blood findings indicated anemia, unprompted infection, increased platelet count, abnormal liver function, and increased CRP levels. Right heart catheterization was performed, with a measured pulmonary artery pressure of 44/22/31 mmHg, a total pulmonary resistance of 5.0 wood units, and a pulmonary vascular resistance of 3.06 wood units. The pulmonary angiography indicated that bilateral pulmonary artery blood flow slowed down, venous reflux slowed down, and distal occlusion of the right pulmonary artery was A10.

After discharge from the cardiology department, the patient continued oral rivaroxaban 10 mg daily for anticoagulation and polysaccharide iron complex 0.3 g daily to correct anemia. However, in January 2022, the patient had a hemoglobin level of 93 g/L↓ and had no significant improvement in anemia, and coagulation suggested a plasma fibrinogen level of 5.3 g/L↑ (2–5 g/L). The patient’s alkaline phosphatase was 516 U/L↑ (reference range: 45–125 U/L) and glutamyltransferase was 112 U/L↑ (reference range: 0–60 U/L). One month after, the patient had spontaneously discontinued the iron polysaccharide complex. The reexamination found that the anemia was significantly aggravated, including hemoglobin: 73 g/L↓ (reference range: 60–110 g/L), RBC count: 3.15 × 10^9^/L↓ (reference range: 3.5–5.5 × 10^9^/L), blood cell ratio measurement: 24.9%↓ (reference range: 37%–50%), MCV: 79 fl (reference range: 80–100 fl), MCH: 23.8pg↓ (reference range: 27–34 pg), and MCHC: 301 g/L↓ (reference range: 320–360 g/L), indicating small cell hypochromic anemia.

**Figure 1:**
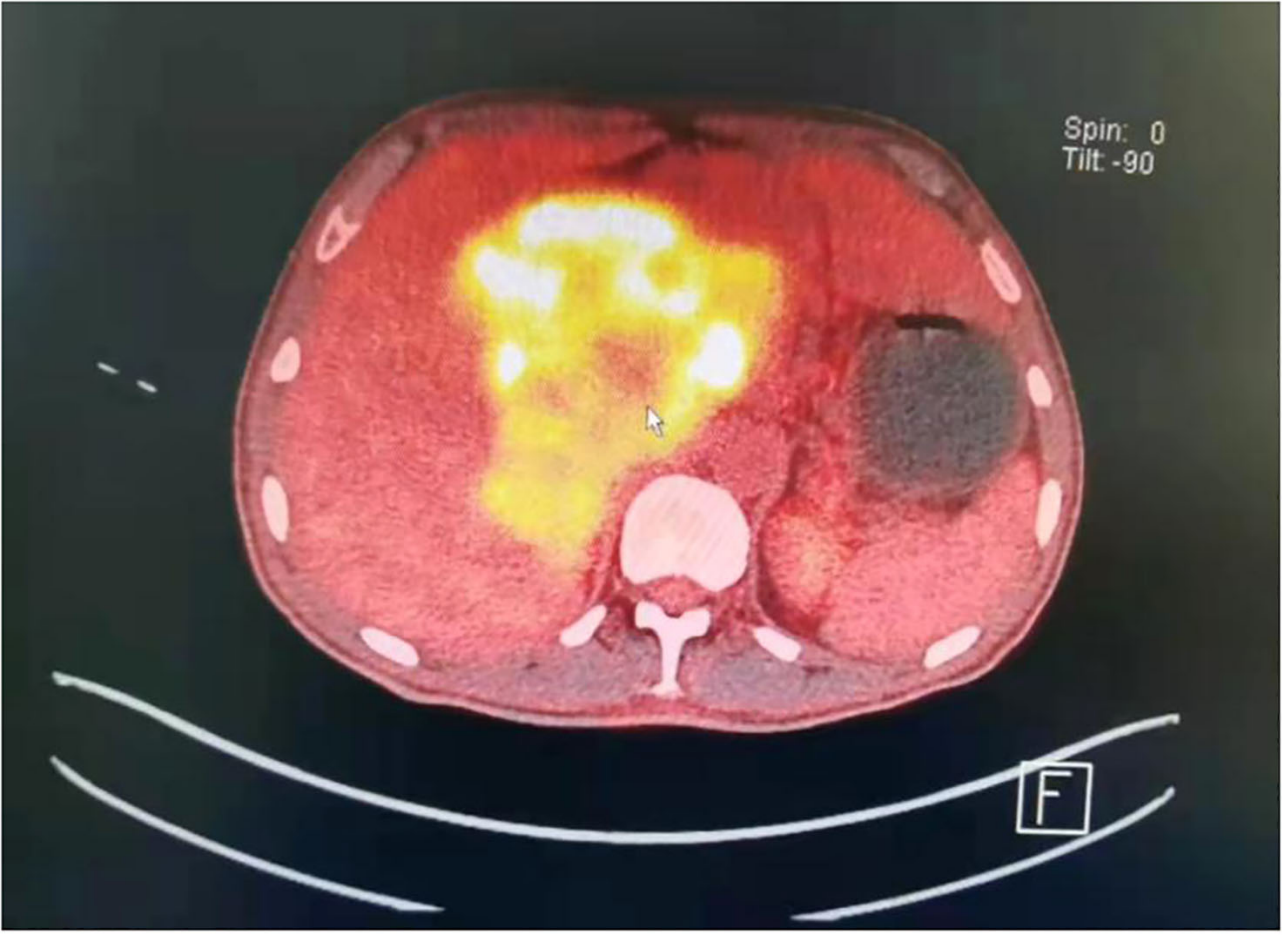
PET-CT, The paraganglioma was in the abdominal and retroperitoneal (liver and pancreatic) area. The interface size of the mass was approximately 148 mm * 100 mm, and the upper and lower diameter was approximately 155 mm.

**Figure 2:**
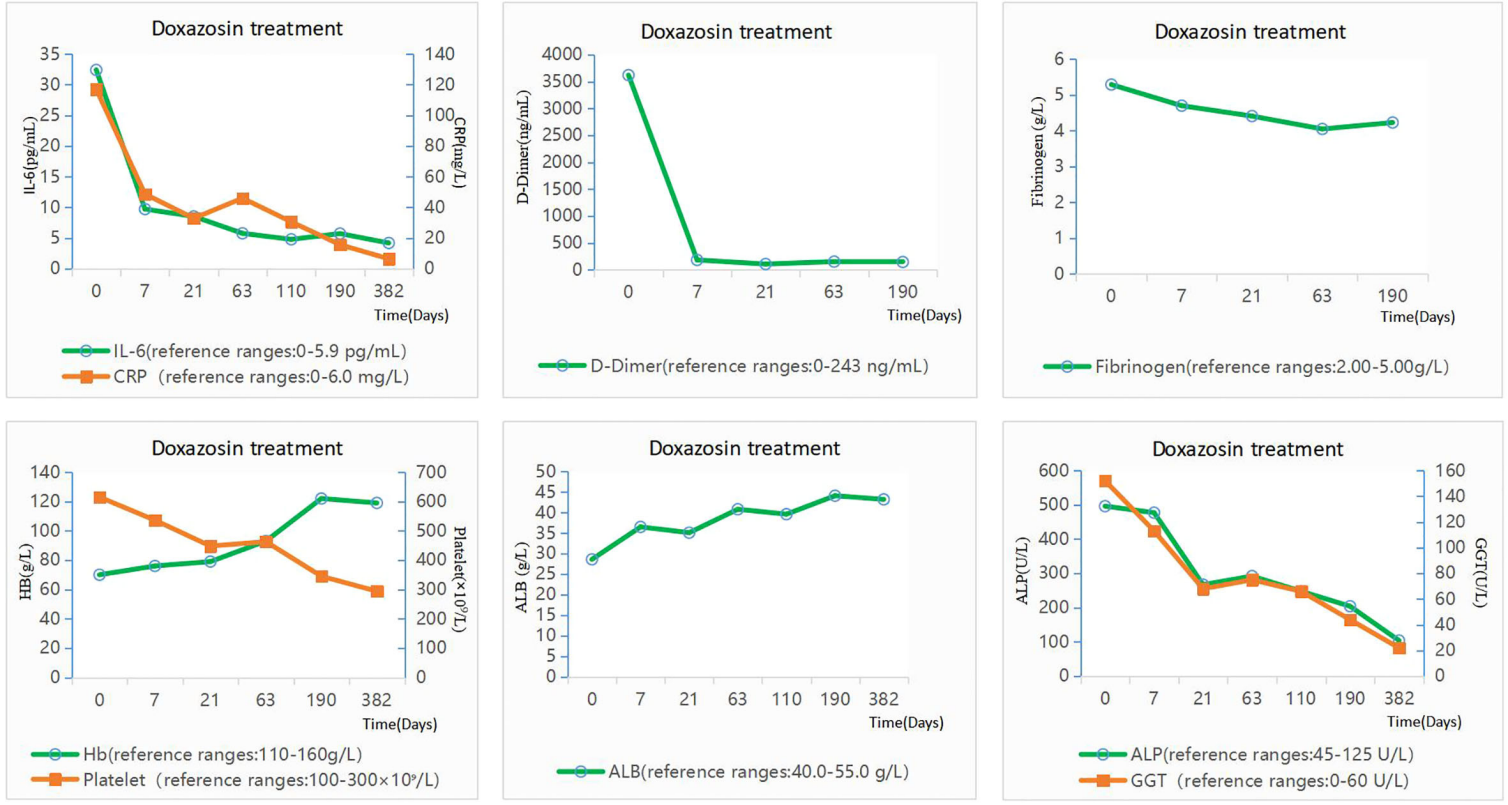
Changes in laboratory data before and after doxazosin treatment. The patient was given doxazosin 4 mg daily.

In February 2022, the patient was hospitalized again due to fever, palpitations, and fatigue. Considering the patient’s previous medical history, the relevant examinations were given. Then, the patient underwent PET-CT examination. 18F-fluorodeoxyglucose (FDG) positron emission tomography/PET-CT showed increased uptake of FDG in the abdominal and retroperitoneal (liver and pancreatic) mass without metastases; the possibility of paraganglioma, and its internal calcification, was considered. The interface size of the mass was about 148mm*100mm, and the upper and lower diameter was about 155mm (**Figure 1**). The demarcation of the mass from the surrounding tissue was unclear, and the surrounding tissue(including: gallbladder, pancreas, stomach, intestinal tube) was pressure-displaced. The patient had the retroperitoneal lymph nodes enlargement which radioactive uptake increased, and the largest lymph node was about 22mm*15mm. The IL-6 and CRP levels were significantly increased, but they showed no abnormalities in IL-1, TNF-α, sCD25, and VEGF. Plasma dopamine was mildly elevated, which is 253.6 pmol/L↑ (reference range: ≤195.7 pmol/L), and this abnormal level may be affected by factors such as food or stress. It did not exceed the normal value twice, so it was not considered as a meaningful clinical change. The urinary dopamine was also not elevated. There were no abnormalities in adrenaline, norepinephrine, 3-methoxytyramine, methoxyepinephrine, methoxynorepinephrine, and Vanillylmandelic acid in peripheral blood and 24-h urine specimens (**Table 1**). At this time, fibrinogen, D-dimer, ALP, GGT, and other indicators were significantly increased (**Figure 2**). Whole exome capture and sequencing of genomic DNA indicated that it was the KIF1B gene mutation, and the chromosomal location was chr1: 10428570, which was a heterozygous gene. This is a mutation at a single-gene rare locus.

**Table 1:** Levels of cytokine, related proteins, serum, and urinary catecholamine before treatment by doxazosin.

	Normal values	Before treatment
IL-6 (pg/ml)(≤5.9)	≤5.9	32.4↑;
IL-1β (pg/m)	≤5.0	≤5.0
TNF-α (pg/m)	≤8.1	7.89
CD-25 (pg/m)	≤6,400	1,896
VEGF(pg/m)	≤160.00	112.11
Plasm		
Dopamine (pmol/L)	≤195.7	253.6↑
Epinephrine (pmol/L)	≤605.4	≤55.5
Norepinephrine (pmol/L)	414.0–4,435.5	2,864.9
3-Mexoxytyramine (nmol/L)	≤0.18	≤0.08
Metanephrine (pmol/L)	≤0.50	≤0.08
Normetanephrine (pmol/L)	≤0.90	0.35
Urine		
Dopamine (nmol/24 )	655.0–3,425.0	1,788.54
Epinephrine (nmol/24 )	8.45–102	≤16.0
Norepinephrine (nmol/24 )	68.9–378.0	179.08
3-Mexoxytyramine (nmol/24 )	≤216	28
Metanephrine (nmol/24 )	≤312	85
Normetanephrine (nmol/24 )	≤382	102
Vanillylmandelic acid (mg/24 )	≤12.0	4.8

↑ indicates that the patient’s IL-6 and dopamine levels were above the normal values before treatment with doxazosin.

Because the patient and his family refused surgery, doxazosin was administered at 4 mg daily for treatment. The anti-inflammatory and antiviral treatment was stopped. The patient’s temperature gradually decreased and returned to normal after 3 days. After 7 days, the levels of IL-6, CPR, ALP, platelets, and GGT decreased significantly, and even fibrinogen and D-dimer returned to normal levels. After 63 days of doxazosin treatment, IL-6 levels became completely normal, albumin (ALB) returned to normal, and anemia was greatly improved. After 190 days of medication, IL-6 continued to have normal levels, and hemoglobin and GGT indicators returned to normal. Meanwhile, the patient’s CRP, ALP, and platelets continued to decline and the patient’s symptoms of fever and palpitations did not reappear. After about 1 year, the patient’s blood indicators were examined again, and all the indicators were normal including IL-6 (**Figure 2**).

## Diagnosis

The patient was febrile due to the increased IL-6 levels caused by the retroperitoneal paraganglioma, but both catecholamines and their metabolites were normal. The patient had a rare single-gene mutation: the KIF1B gene.

## Treatment and outcome

Antibiotic and antiviral therapy were stopped. The patient was given doxazosin 4 mg daily since 4 February 2022. After 3 days of taking doxazosin, the body temperature of the patient gradually decreased and returned to normal range. The heart rate decreased to approximately 90 bpm. After 7 days, the levels of IL-6, CPR, ALP, platelets, and GGT decreased significantly, and even fibrinogen and D-dimer returned to normal levels. After 63 days of doxazosin treatment, IL-6 level was completely normal, albumin returned to normal levels, and anemia was greatly improved. After 190 days of medication, IL-6 continued to have normal levels, and hemoglobin and GGT indicators returned to normal. Meanwhile, the patient’s CRP, ALP, and platelets continued to decline and the patient’s symptoms of fever and palpitations did not reappear. Several days ago, after 1 year onset, the patient again underwent a blood test. Almost all blood indexes were in the normal range including IL-6 (**Figure 2**).

## Discussion

Pheochromocytoma (PCC) and paraganglioma (PGL) occur in 2 to 8 per million people (1). Paragangliomas (PGLs) are rare neuroendocrine tumors that arise from chromaffin cells of extra-adrenal paraganglia usually with persistent or intermittent hypertension accompanied by headache and/or palpitations (1). This case had symptoms of fever and palpitations, without hypertension. Cases of paraganglioma presenting with unexplained fever as the main symptom were very rare. In addition to secreting large amounts of catecholamines, paragangliomas can also secrete a variety of cytokines including IL-6 that mediates inflammatory response and induces the production of acute phase proteins such as CRP and fibrinogen (7). The reasons for the increase in IL-6 levels might be indirect results of high levels of circulating norepinephrine, and sometimes rarely secreted by the tumor (8). In our case, a significant increase in IL-6 was only seen from biochemical assays, while blood and urine catecholamines or their metabolites were in the normal range. The increase in IL-6 was secreted by the tumor directly. Long-term IL-6 secretion resulted in changes in laboratory tests (CRP, fibrinogen, D-dimer, platelets, ALP, and GGT) and symptoms (fever, palpitation, fatigue, and anemia) in our case. We considered that elevated IL-6 may be associated with retroperitoneal paraganglioma secreting with IL-6 for the patients. A total of 42 cases of pheochromocytoma or paraganglioma with elevated IL-6 have been reported (9). As in our case, only IL-6 was elevated, and no abnormalities in catecholamine and its metabolites have not been reported.

The 5-year survival rate of PPGL is as high as 50% to 70% (10), which means the disease can be considered a chronic disease in some conditions. Moreover, regardless of PPGL type (benign or malignant), long-term survival could not be achieved even after surgical resection, and the recurrence rate is high (11). For some tumors that cannot be safely removed completely, other treatments, such as oral drugs, are required besides surgery to control bio-active substance secretion and the progressive growth of tumor. In our case, the large tumor lying in the abdominal cavity and retroperitoneum invaded peripheral tissues including pancreas and intestines. After assessing the risk of operation and having no significant changes in the size of the mass by comparing with CT images 10 years ago, oral drugs were the first and safest choice for the patients. Doxazosin was selected for the patients because alpha-blockers are currently the most common oral drugs before and after surgery for paraganglioma.

Different types of gene mutations in PPGL patients are directly related to the mass location, and possibly to metastasis and the variety of clinical manifestations. To date, over 20 gene mutations, including EGLN1/PHD2, KIF1B, and IDH1 genes, are thought to potentially contribute to the pathogenesis of PPGL (1). KIF1B gene mutation was noticed in recent reports, which suggested that KIF1B was the second most frequently mutated gene and often combined with other gene mutations in PPGL cases (12). To date, only two cases with KIF1B single gene mutation in PPGL were reported (in 2017 and 2022), both with elevated levels of blood and urine epinephrine and norepinephrine (12, 13). The whole-exome capture and sequencing of the genomic DNA of our patient only showed a heterozygous mutation in the coding site of KIF1B gene (Coding: NM_015047.3:c.4660G >C, Mutation: p.Val1554Leu; chromosomal location was chr1: 10428570). The single mutation at this locus of KIF 1 B has not been previously reported.

There is the question that the tumor had been existing silently over 10 years. What then triggered the eruption of the “silent volcano”? Reviewing the history carefully, we found that the patients had been treated with betamethasone for a large area of eczema 4 months starting in September 2021, which was 4 months before the appearance of fever in February 2022. Corticosteroids are one of the reasons that could cause the paraganglioma to “awaken” from its silent state (1, 4).

## Conclusions

This case reports a rare case of retroperitoneal paraganglioma, which suddenly presented with unexplained fever after 10 years of tumor silence. The case only showed a significant increase in the level of IL-6, and there were no clinically meaningful increases in other catecholamines and metabolites. After the patient was treated with the alpha-blocker doxazosin, the level of IL-6 dropped significantly, the body temperature returned to normal, and the heart rate also improved. Paraganglioma that only secretes IL-6 as the main manifestation is extremely rare at present, which suggests that clinicians should understand the secretion mode of this rare paraganglioma and the clinical symptoms of patients, and should make a differential diagnosis in patients with unexplained fever, as well as consider the possibility of the existence of paraganglioma. The application of doxazosin may become a long-term and effective non-surgical means of treating paraganglioma, and provide a new therapeutic direction for the treatment of paraganglioma. In the gene exon sequencing of this case, it was found that the patient had a rare KIF1B single-gene mutation. Although we could not clearly prove its pathogenicity, we provided some evidence for future clinical and genetic data, which may be helpful for future pathogenicity studies of this gene in PPGL disease.

## Data availability statement

The datasets presented in this study can be found in online repositories. The names of the repository/repositories and accession number(s) can be found in the article/supplementary material.

## Ethics statement

The studies involving humans were approved by The Medical Ethics Committee of the Second Affiliated Hospital of Harbin Medical University. The studies were conducted in accordance with the local legislation and institutional requirements. The participants provided their written informed consent to participate in this study. Written informed consent was obtained from the individual(s) for the publication of any potentially identifiable images or data included in this article. Written informed consent was obtained from the participant/patient(s) for the publication of this case report.

## Author contributions

CW is responsible for data collection, collation, literature review and article writing, the conception of the article, and participated in the whole treatment process of the patients. CC is the corresponding author of this case report. CC was responsible for the diagnosis and treatment of the case, and the conception of this article. CC and CW have discussed the article repeatedly. MG is responsible for caring for the patients in the case and observing the patient’s condition changes. SZ is responsible for the format proofreading of this paper. All authors contributed to the article and approved the submitted version.
